# Eight key rules for successful data‐dependent acquisition in mass spectrometry‐based metabolomics

**DOI:** 10.1002/mas.21715

**Published:** 2021-06-18

**Authors:** Emmanuel Defossez, Julien Bourquin, Stephan von Reuss, Sergio Rasmann, Gaétan Glauser

**Affiliations:** ^1^ Laboratory of Functional Ecology, Institute of Biology University of Neuchâtel Neuchâtel Switzerland; ^2^ Waters Corporation Wilmslow UK; ^3^ Laboratory of Bioanalytical Chemistry, Institute of Chemistry University of Neuchâtel Neuchâtel Switzerland; ^4^ Neuchâtel Platform of Analytical Chemistry University of Neuchâtel Neuchâtel Switzerland

**Keywords:** cycle time, DDA, exclusion list, mass window, precursor selection, Q‐TOF, tandem mass spectrometry

## Abstract

In recent years, metabolomics has emerged as a pivotal approach for the holistic analysis of metabolites in biological systems. The rapid progress in analytical equipment, coupled to the rise of powerful data processing tools, now provides unprecedented opportunities to deepen our understanding of the relationships between biochemical processes and physiological or phenotypic conditions in living organisms. However, to obtain unbiased data coverage of hundreds or thousands of metabolites remains a challenging task. Among the panel of available analytical methods, targeted and untargeted mass spectrometry approaches are among the most commonly used. While targeted metabolomics usually relies on multiple‐reaction monitoring acquisition, untargeted metabolomics use either data‐independent acquisition (DIA) or data‐dependent acquisition (DDA) methods. Unlike DIA, DDA offers the possibility to get real, selective MS/MS spectra and thus to improve metabolite assignment when performing untargeted metabolomics. Yet, DDA settings are more complex to establish than DIA settings, and as a result, DDA is more prone to errors in method development and application. Here, we present a tutorial which provides guidelines on how to optimize the technical parameters essential for proper DDA experiments in metabolomics applications. This tutorial is organized as a series of rules describing the impact of the different parameters on data acquisition and data quality. It is primarily intended to metabolomics users and mass spectrometrists that wish to acquire both theoretical background and practical tips for developing effective DDA methods.

**Abbreviations**
AGC: automatic gain control
CID: collision‐induced dissociation
DDA: data‐dependent acquisition
DIA: data‐independent acquisition
HRMS: high‐resolution mass spectrometry
MRM: multiple reaction monitoring
MS: mass spectrometry
MS/MS: tandem mass spectrometry
QqQ: triple quadrupole
Qq‐FT‐ICR: quadrupole‐Fourier transform ion cyclotron resonance
Q‐TOF: quadrupole‐time‐of‐flight
TOF: time‐of‐flight
UHPLC: ultrahigh performance liquid chromatography

## 1 INTRODUCTION

Over 3.5 billion of years of evolution, natural selection has generated an overwhelming array of molecular entities forming the backbone of the ever‐growing tree of life. This myriad of compounds, crafted from 27 out of the 90 elements present on Earth and produced by all living organisms, from archaea to whales, represent millions of potential functional entities (Alseekh & Fernie, 2018; Aversa et al., 2016; Frieden, 1972; Xie et al., 2015). Generated at the crossroad of any individual's genome, its physiology, and its environmental adaptation, this massive chemodiversity provides the raw material for both fundamental and applied biological research. For instance, many of the molecules produced by plants bear pharmacological properties (e.g., anticancer, antibacterial, antiviral, analgesic, anti‐inflammatory or antitumor), making them an invaluable source of medicines (Bruneton, 1995; Newman & Cragg, 2020; Veeresham, 2012). Since the late 1990s, when the term “metabolome” was first coined to define the collection of small‐molecular‐weight molecules (typically <1500 Da) produced by a given organism, metabolomics has emerged as a pivotal approach to uncover the molecular structure of biological systems (Fiehn, 2002). This rapidly evolving technological approach, notably via the development of mass spectrometry (MS) workflows, has already generated abundant information on the nature of biochemical processes and functions across scales: from cells to individual organisms, to ecosystems (Chomel et al., 2016; Tanentzap et al., 2019), as well as for the development of more accurate medical and drug discovery programs (Castro‐Perez, 2007; Thomford et al., 2018). Nonetheless, today, to exhaustively detect, characterize, and quantify the entire metabolome of biological systems remains a major analytical challenge in chemical biology research (Fiehn, 2002; Patti et al., 2012).

Of all the different techniques used for metabolomic analyses, mass spectrometry is among the preferred tools, due to its high selectivity and sensitivity (Cajka & Fiehn, 2016). Mass spectrometry‐based metabolomics can be broadly divided into two groups: targeted or untargeted (Roberts et al., 2012). Targeted analysis involves multiplexed analysis of a set of defined metabolites, generally using multiple reaction monitoring (MRM) with low‐resolution tandem mass spectrometers (Lu et al., 2008). This type of analysis facilitates metabolite identification and quantification, and minimizes the risk of false annotation. A major drawback of the targeted approach, however, is the limited metabolome coverage and the impossibility to perform retrospective data analysis. In contrast, untargeted analysis aims to detect as many metabolites as possible in a single or integrated analysis and offers the potential to discover new biomarkers without pre‐existing knowledge (Ribbenstedt et al., 2018). Typically, for untargeted metabolomics, (ultra)‐high pressure liquid chromatography is combined with high‐resolution mass spectrometry (HRMS), such as when using quadrupole‐time‐of‐flight (Q‐TOF) and Orbitrap instruments.

Several approaches have been proposed to perform mass spectrometry‐based untargeted metabolomics: full scan, data‐independent acquisition (DIA), or data‐dependent acquisition (DDA) (Fenaille et al., 2017). In full scan mode, only one MS function without induced fragmentation is acquired to generate ions of the molecular species, adducts and in‐source fragments. Despite the low level of spectral information and metabolite identification provided by the full scan acquisition mode, several untargeted metabolomics studies still use this approach, probably due to its simplicity in terms of acquisition, data processing, and its high performance for discriminating biological samples (e.g., Clancy et al., 2018; Marr et al., 2021). To improve data quality and metabolite annotation rate, fragmentation data using DIA or DDA can be added to the full scan mode. In conventional DIA, including the so‐called MS^E^, MS^ALL^, or MS‐AIF, one function is set at low collision energy and is equivalent to a full scan analysis, whereas a second function is set at higher collision energy to generate molecular fragmentation (Plumb et al., 2006; Wrona et al., 2005). The main advantage of DIA is that, thanks to its fast acquisition rate, no undersampling of some peaks occurs allowing fragmentation of all precursor ions. However, in DIA, it is not possible to deduce the physical relationship between multiple precursor ions and their fragments. Therefore, subsequent mass spectral deconvolution relies uniquely on chromatographic retention time differences and on the quality of time alignment by the processing software (Schrimpe‐Rutledge et al., 2016). This often results in very complex fragmentation spectra and poor match between precursors and fragments (van der Laan et al., 2020). To enhance selectivity, improved DIA methods have been proposed such as the SWATH or SONAR acquisition methods, in which the quadrupole analyser of a Q‐TOF mass spectrometer is stepped or ramped across a mass range of interest (Bonner & Hopfgartner, 2019; Gethings et al., 2017; Gillet et al., 2012). By reducing the ion transmission window for the first quadrupole (Q1), for example, from 1000 Da to 20–30 Da at a given time, the probability that several precursor ions are simultaneously fragmented is proportionally reduced. Alternatively, an ion mobility device may be used in combination with the high‐resolution mass spectrometer to obtain cleaner spectra and reduced interferences (Paglia & Astarita, 2017). Still, these DIA techniques are not always capable of reliably revealing the connection between the precursor and the fragment ions.

**Figure 1:**
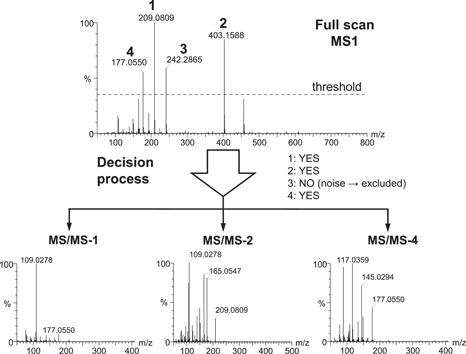
Principle of data‐dependent acquisition. The mass spectrometer first performs a full scan MS1 survey. Only the ions that meet certain defined criteria are further selected for MS/MS

By contrast, DDA offers the possibility to acquire real MS/MS spectra by selecting precursor ions from a full scan analysis for further fragmentation, based on real‐time evaluation of MS data by the software. DDA thus has the potential to significantly improve metabolite annotation, notably via in silico fragmentation tools, by providing cleaner spectra compared to other acquisitions modes (Guo & Huan, 2020). In a DDA experiment, the fragmentation is only performed on the MS signals that meet specified, user‐guided criteria (Figure 1). These criteria are diverse and relatively complex but represent critical steps for optimizing the coverage of the metabolome of the biological system under investigation. Moreover, several compromises, such as in terms of number of MS/MS spectra that can be acquired versus the speed of chromatography, must be made (Ten‐Doménech et al., 2020). A number of studies have reported on how to increase coverage using sophisticated approaches such as time‐staggered precursor lists or data set‐dependent acquisition (Broeckling et al., 2018). However, given that DDA parameter choice is so important for obtaining high data quality, the small number of systematic studies that focus on the effects of the different DDA settings is striking. We have observed large discrepancies, and occasionally idiosyncratic use, in the DDA parameters published in the literature. Besides, a significant proportion of publications simply omit to report the essential parameters needed to reproduce a particular DDA experiment, thus impairing proper evaluation of the methods (see Supporting Information, for an example of a correct description of a DDA method).

To overcome this gap in the literature, we here present a tutorial which aims to provide guidelines on how to choose and optimize the technical parameters essential to perform any DDA metabolomics experiments. We describe which effects these parameters have on the data outcome, and how they are interrelated. This tutorial is not a comprehensive review on the applications of DDA in metabolomics, but rather a compilation of data, observations, and recommendations resulting from our own experience, discussions with colleagues and literature searches. This tutorial is organized as a list of eight rules that we consider to be of primal importance for successful DDA analyses. We trust that this tutorial can help users make appropriate decisions when setting up a DDA method, which will ultimately maximize MS/MS data performance for innovative metabolomics workflows.

## 2 EIGHT RULES FOR SUCCESSFUL DDA ANALYSES

Although DDA can in theory be performed on any tandem mass spectrometer such as the triple quadrupole (QqQ), quadrupole‐linear ion trap (QqLIT), Q‐TOF, Orbitrap (LTQ‐Orbitrap, Q‐Exactive), and Qq‐FT‐ICR, in practice Q‐TOFs or Orbitraps are predominantly employed since only these two technologies combine sufficient resolution and fast enough acquisition frequency to facilitate metabolomics analyses. In the following section, we thus focus on these two types of hybrid instruments. The parameter values and data presented are derived from measurements performed on Synapt XS (Waters) and TripleTOF 6600 (AB Sciex) Q‐TOFs, as well as on a Q‐Exactive HF (Thermo Fisher Scientific). However, the presented concepts are generic and should be applicable to any Q‐TOF or Orbitrap instrument with minimal adjustments.

### 2.1 Rule 1: Set appropriate scan time and maximum number of MS/MS per cycle

**Figure 2:**
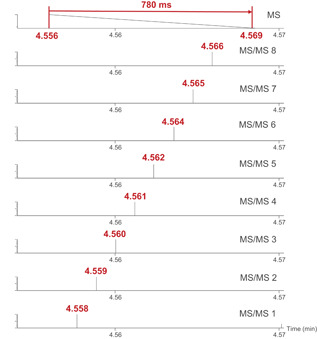
Illustration of the experimental determination of the total cycle time. In this example where one full scan MS1 and eight MS/MS are acquired, the time between two full scan events corresponds to 4.569–4.556 min = 0.013 min = 780 ms [Color figure can be viewed at wileyonlinelibrary.com]

In DDA, the instrument acquires a full scan MS1 and, when certain criteria (described thereafter) are met, performs a specified number of MS/MS acquisitions on the most intense ions before switching back to full scan MS1. According to the literature, there exists great heterogeneity in the scan times selected for MS1 and MS/MS acquisitions, typically ranging as widely as 20–800 ms (Andrews et al., 2011; Pezzatti et al., 2020), as well as the maximal possible number of MS/MS to be recorded (typically between 3 and 20). A longer scan time definitely provides a gain in sensitivity, albeit at the expense of an increase in total cycle time, that is, the time needed for switching from one to the next full scan MS1 acquisition (Figure 2). A higher number of subsequent MS/MS also increases the total cycle time. Since the total cycle time determines the frequency (scans/s), it should be carefully adapted so that enough data points per chromatographic peak (i.e., at least 7–8) can be obtained. However, the time that the MS takes to complete a cycle of MS1 and MS/MS acquisitions cannot simply be calculated by summing the MS1 and MS/MS acquisition times specified in the method, but should be experimentally determined (Figure 2). Indeed, there exist additional delay times during which (i) the instrument switches from one function to another (the interscan delay), and (ii) the data‐dependent computational processing performed by the software selects the top ions from the MS1 acquisition for further fragmentation. For instance, we have experimentally measured that, on the Synapt XS Q‐TOF (Waters), the sum of the interscan delay (14 ms) and the computational process between each event takes on average 31 ms. On the TripleTOF 6600 (AB Sciex), we also measured a delay time of 31 ms. This means that for a method with one full scan MS1 and nine MS/MS, these instruments will spend up to 310 ms per cycle without acquiring data. This time lag can be significant in combination with ultrahigh performance liquid chromatography (UHPLC), which has become standard in metabolomics and in which peak widths are typically 4–6 s (Johnson et al., 2013). In such a situation, the acquisition of 7–8 data points per peak along with a large number of MS/MS restricts the available scan time and thereby limits overall sensitivity.

**Figure 3:**
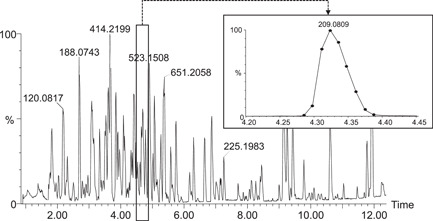
Full scan MS1 chromatogram of a plant extract analysed by DDA with 1 MS survey and max. eight MS/MS. Inset: extracted ion chromatogram at *m/z* 209.0809 from the full scan MS1. The total cycle time is constantly 780 ms (all available MS/MS functions were used at all times) and enables nine data points over the 6‐s‐wide chromatographic peak. Data were acquired in continuum mode on a Synapt XS (Waters)

In our methods using the last‐generation Synapt XS Q‐TOF, we generally set scan times of 100 ms for MS1 and 50 ms for MS/MS and allow a maximum of eight subsequent MS/MS functions. This results in a total cycle time of 780 ms (500 ms acquisition + 280 ms delay) in profile mode and enables at least eight data points per chromatographic peak (Figure 3). Other authors have used identical set ups on other systems (Uka et al., 2017). Slower instruments might permit only a smaller number of MS/MS events or require slower chromatography to enable enough data points per peak. Centroiding data may also increase the cycle time. In any case, it is simply unrealistic to acquire up to 100 MS/MS in combination with fast chromatography as occasionally advertised by certain manufacturers because the delay time alone would take about 3 s. It should also be noted that the cycle time at a given moment may be shorter than the maximal possible cycle time if fewer MS/MS than the maximum enabled are acquired during this cycle. This may lead to erroneous interpretation of the cycle time and for this reason, it is advisable to calculate the total cycle time using a very low or null threshold to make sure that all available MS/MS functions are acquired at all times.

Finally, on the Orbitrap mass analyser, the MS and MS/MS events are controlled by both a maximum ion injection time and an automatic gain control (AGC) target value, depending on which parameter is reached first (Kalli et al., 2013). This can result in variable scan times for both MS1 and MS/MS functions. Also, unlike Q‐TOFs, the scan time and the delay time on the Orbitrap increase with increasing resolution and a compromise must be made between frequency and resolution. Accordingly, most DDA methods on the Orbitrap typically use a higher resolution in MS1 (*R* = 35,000–70,000) than in MS/MS (*R* = 17,500–30,000). Although the average delay time per cycle is slightly longer on the Orbitrap (39–57 ms depending on the resolution) than on TOFs (31 ms, see above) because of the computation intensive Fourier transformation, similar cycle times can be obtained with both technologies. For instance, using a method with one full scan MS1 and eight MS/MS functions on a Q‐Exactive HF, we have experimentally measured cycle times of 600, 715, and 880 ms for MS1/MS2 resolutions set to 45,000/15,000, 60,000/30,000, and 120,000/30,000, respectively.

### 2.2 Rule 2: Define the proper threshold for switching to and back from MS/MS

The signal threshold at which the instrument will switch from full scan MS1 to MS/MS is one of the most important parameters for DDA. On the one hand, if the threshold is set too high, many peaks detected in the full scan MS1 acquisition will not be fragmented, which will result in limited MS/MS coverage. On the other hand, if set too low, MS/MS will be performed on (i) low intensity signals giving rise to low quality MS/MS spectra, often insufficient for metabolite assignment, and (ii) noise signals giving rise to “useless” MS/MS spectra. The threshold strongly depends on the signal intensity and background noise level of the mass spectrometer, which can differ a lot according to the manufacturer and instrument type, as well as on solvent purity, memory effects of the column, condition of the source, and so on. Furthermore, some high‐resolution mass spectrometers can be operated at variable resolutions and the threshold may vary according to the specified resolution. Indeed, the higher the resolution on Q‐TOF instruments, the lower the absolute signal intensity. Noteworthily, the threshold value is also closely associated with the peak exclusion list, which permits to decrease the threshold while preventing (the excluded) background ions to be selected for MS/MS fragmentation (see Rule 3 below). In our DDA methods, we set signal thresholds that are about 5–10 times lower than the highest signals present in the background noise. Finally, another key aspect is to define the return to full scan MS1 after the MS/MS acquisition. There are basically two means to achieve this: (i) by setting a constant accumulation time for MS/MS (e.g., 50 ms for each MS/MS followed by return to MS1), or (ii) by setting an accumulation target intensity (e.g., 10,000 counts for each MS/MS followed by return to MS1). We recommend when possible to preferentially define a constant accumulation time rather than intensity, for reasons related to the total cycle time (see Rule 1) which may vary widely when a target intensity is defined.

### 2.3 Rule 3: Utilize a recent exclusion list and determine exclusion mass tolerance and exclusion time

**Figure 4:**
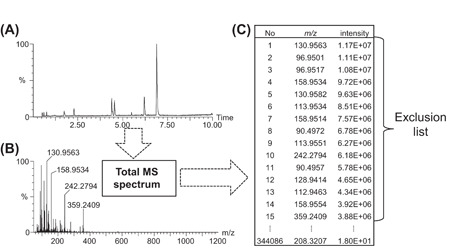
Schematic representation of the creation of an exclusion list from a blank analysis. First, a blank sample is analysed. (A) The corresponding base peak intensity chromatogram. Second, a total MS spectrum is generated from the blank chromatogram (B). Third, a list of all detected peaks in this total MS spectrum is exported and sorted according to intensity (C). The exclusion list is created with the most intense background ions

A static exclusion list is a number of features (characterized by their *m/z* or *m/z* vs. retention time) which should never be selected for MS/MS even if they exceed the defined threshold. Having an exclusion list is crucial for enabling the system to select ions whose intensity is smaller than those of the most intense background ions. Since the noise in mass spectrometry can vary quantitatively and qualitatively over time, we recommend to generate a new exclusion list for every batch of analyses. There are several ways to create exclusion lists, but we propose here the following simple procedure based only on *m/z* ratios: first run 4–5 blank solvent samples, then generate a total mass spectrum over the last chromatogram, and finally sort the detected ions by order of intensity and keep only the most intense ions for the exclusion list (Figure 4). With this approach, retention times are not taken into account and the exclusion is active across the whole chromatogram. Thus, it is particularly efficient at excluding background interferences that elute as baseline noise or broad peaks but less efficient on ghost peaks. Generally, a rule of thumb is to exclude the background ions which have an equal or higher intensity than the threshold intensity set for MS/MS switch. Excluding less ions would cause the preferential selection of background ions over signals of interest, whereas excluding more ions could increase the risk of accidentally omitting certain ions of interest in subsequent analyses (see also the paragraph on mass tolerance below). The number of excluded precursor ions is highly correlated to the instrument sensitivity. For instance, on the Synapt G2 Q‐TOF, which is an old‐generation instrument bearing low sensitivity, we exclude about 10 ions in positive electrospray when operated in resolution mode. In contrast, on the newer Synapt XS Q‐TOF which displays much higher sensitivity in the same configuration, we exclude about 200 ions. Naturally, other factors such as the quality of the solvents used for chromatography, the memory effect of the column, the condition of the source, and the acquisition mode (profile or centroid) may also have an impact on the number of ions that need to be excluded.

**Figure 5:**
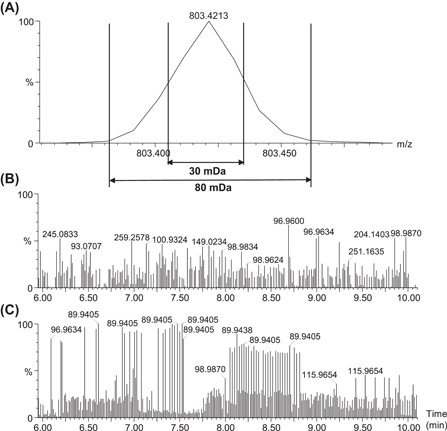
Illustration of the effect of the mass tolerance window around the excluded ions. (A) Zoom on a peak at *m/z* 803.42. An 80 mDa but not 30 mDa window is sufficient to entirely cover the peak. (B) MS/MS first trace of a blank sample with the tolerance window set at 80 mDa. (C) MS/MS first trace of a blank sample with the tolerance window set at 30 mDa. Redundant intense peaks at *m/z* 89.94 are visible along the chromatogram and indicate that the precursor ion leading to the *m/z* 89.94 fragment was not constantly excluded due to a too narrow mass tolerance window. Data were acquired in continuum mode on a Synapt XS (Waters) operated in “resolution” mode (resolution ca. 23,000 at *m/z* 556)

Another key aspect of the static exclusion list is the mass tolerance in mDa (also referred to as mass window) around the exact mass of excluded precursor ions. Regardless of whether data are acquired in profile or centroid modes, this tolerance depends mostly on the MS resolution and should be determined for a specific instrument based on the same concept as that of mass extraction windows for HRMS quantitative methods (Glauser et al., 2016; Vereyken et al., 2018). If the tolerance is set too low, redundant “spikes” of yet excluded ions will still appear in the MS/MS functions because the edges of high intensity MS peaks will still be accounted for (Figure 5). On the other hand, a too high tolerance will prevent the selection of certain isobaric compounds and create significant gaps in the covered mass range. For example, setting a list of 1000 excluded precursors with a 100 mDa tolerance could cause a potential loss of up to 100 Da over the whole mass range. On the Synapt XS Q‐TOF, we have experimentally determined optimal tolerances of 100, 80, 30, and 20 mDa for sensitivity (resolving power of ca. 13,000 at *m/z* 556), resolution (resolving power of ca. 22,000 at *m/z* 556), high resolution (resolving power of ca. 45,000 at *m/z* 556) and enhanced resolution (resolving power of ca. 63,000 at *m/z* 556) modes, respectively. On the Sciex TripleTOF 6600, the adequate mass tolerance is 50 mDa for a resolving power of ~35,000 at *m/z* 956. It should be noted that the resolution on TOF instruments is relatively stable over the whole mass range, meaning that the peak width increases with the *m/z* ratio. Thus, in theory, heavier ions would need a larger tolerance than lighter ions. This phenomenon may even be exacerbated on Orbitrap machines as resolution decreases with increasing *m/z*. At this stage, we are unaware of software solutions which take this aspect into consideration, and which would enable the user to set variable mass tolerances over the *m/z* range.

In addition to the static exclusion list, a dynamic exclusion list enables the user to define an exclusion time during which a specific precursor that had been selected for MS/MS is omitted from being selected again. We found that there is actually a high discrepancy in the values used for this parameter in the literature, some of which can be as short as 0 s and others as long as 30 s or even for the rest of the analytical run. Setting the exclusion time too low will result in repeated MS/MS acquisitions of the same precursor ions over the same chromatographic peak to the detriment of the less intense ions, thereby reducing MS/MS coverage. Conversely, setting it too long (e.g., 30 s) will prevent the switch to MS/MS for closely eluting isomers, which are frequent in the metabolomics analysis of biological samples. We advise to use a trade‐off approach that ranges between these two extreme situations. For UHPLC separations with peak widths of ca. 4‐6 s, we usually set an exclusion time of 1.5–2 s. This enables us to acquire a second MS/MS at the top of the peak to get more intense fragments and better spectral quality. Alternatively, one may set an exclusion time which is slightly longer than the peak width at the base so that no redundant MS/MS spectra are acquired.

Finally, it is possible to perform iterative automated precursor ion exclusion using repeated injections of the same sample. After a first round of DDA, a list of all selected precursor ions is created and imported as exclusion list in the method for a second DDA run, and so on. This process can be indefinitely repeated with new lists merged with the lists generated from the previous runs, but the best compromise was found to be five rounds of exclusion (Zhang, 2012). The advantage of this approach is that it maximizes the number of unique MS/MS spectra. However, the time required to measure a single sample may be prohibitive in the case of large sample batches.

### 2.4 Rule 4: Utilize an inclusion list (optional)

An inclusion list, a list of desired precursor ions that are preferentially selected for MS/MS, is rare in untargeted metabolomics studies, which by definition aim for an unbiased coverage of all metabolites. However, similarly to the automated precursor ion exclusion method mentioned above, inclusion lists may be used in a sequential manner to increase MS/MS coverage (Hoopmann et al., 2009). Here again, the information obtained from previous runs is used to guide the acquisition of MS/MS spectra in subsequent analyses. To do so, a full scan analysis is first acquired and an inclusion list is generated using a feature detection software. Inclusion lists consist of features characterized by *m/z* values and narrow retention time windows. Only the detected features will then be selected for MS/MS in the next run. Inclusion lists can even be combined with exclusion lists for in‐depth MS/MS coverage (Cho et al., 2021).

### 2.5 Rule 5: Adjust the collision energy for MS/MS fragmentation

**Figure 6:**
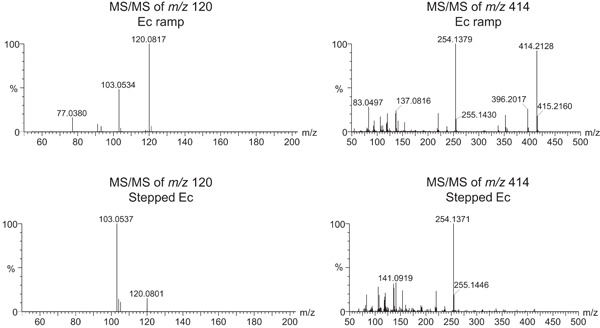
Examples of MS/MS spectra for two representative metabolites of a plant extract using a ramp of collision energy weighted by *m/z* (5–40 V at *m*/*z* 50 and 20–70 V at *m*/*z* 1200) or a stepped collision energy (10‐20‐30‐40‐50 V). In both cases, the ramped collision energy provides more informative fragments than the stepped collision energy

The ability to annotate metabolites is directly dependent on the quality of MS/MS fragmentation. Ideally, fragmentation conditions should be selected so that all molecules within a run fragment neither too strongly, nor too weakly. Several options are available for fragmenting precursor ions using collision‐induced dissociation (CID) in the collision cell: (i) a unique fixed collision energy (e.g., 25 V), (ii) stepped collision energies (e.g., 10, 20, 30, 40, and 50 V), and (iii) a collision energy ramp (e.g., 10–50 V). In DDA, an additional possibility is to set mass‐dependent or retention time‐dependent collision energies. Indeed, small molecules generally require lower collision energies than big molecules. Our tests have revealed that, on the Synapt XS Q‐TOF, a wide ramp of collision energy weighted by *m/z* gives the best overall fragmentation spectra for diverse metabolites (Figure 6). Specifically, we apply a ramp of 5–40 V at *m/z* 50, 12.5–55 V at *m/z* 625, and 20–70 V at *m/z* 1200. These values are indicative and optimal values for a given instrument will depend on the collision cell geometry, the collision gas pressure and type (Ar, N_2_, etc.), or the polarity (negatively charged ions usually require less energy than positively charged ions). We therefore recommend to optimize collision energies on a set of compounds with diverse range of *m/z* and structures.

### 2.6 Rule 6: Adjust the quadrupole or ion trap isolation window for precursor selection

**Figure 7:**
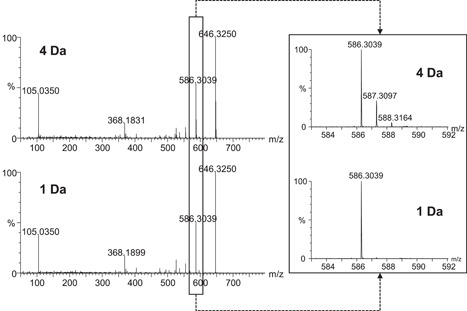
Examples of MS/MS spectra with the quadrupole set at 4 or 1 Da isolation windows. The precursor ion is *m/z* 646 in both cases. Inset: zoom on the fragment at *m/z* 586.3039. Isotopes are present with a 4 Da window but not with a 1 Da window

The quadrupole or linear ion trap of hybrid high‐resolution mass spectrometers such as Q‐TOFs and LTQ‐Orbitrap serve as mass filters during precursor selection. Their mass resolution window can be modulated and this has an impact on MS/MS spectra. The isolation window may be wide (typically 4 Da window), intermediate (2 Da), or narrow (1 Da) (Allard et al., 2016; Kalli et al., 2013). In the wide mode, the 4 Da window is generally set at −0.5/+3 Da around the precursor mass, so that the first three isotopologues are included in the MS/MS spectrum (Figure 7). In the narrow mode, a −0.5/+0.5 Da window is set around the precursor mass, and the information provided by the isotopologues is lost (Figure 7). Naturally, the narrow mode provides better selectivity but slightly lower sensitivity (1.5–2‐fold on our instrument). Furthermore, the loss of isotopic patterns can have a negative impact on molecular formula assignment, as spectral accuracy (i.e., the accurate measurement of isotopic distributions (Glauser et al., 2013)) is an important factor for the determination of elemental compositions (Kind & Fiehn, 2007). Altogether, there is thus a trade‐off between selectivity, sensitivity, and spectral information that should be considered when setting the isolation window.

### 2.7 Rule 7: Apply filters for precursor selection

Depending on the manufacturer, different filters for precursor selection may be available. Here we discuss only two which in principle should be common to all types of instruments. First, an isotope exclusion function (also called monoisotopic precursor selection) should be activated, so that the mass spectrometer does not spend time to measure different isotopologues of the same metabolite. Second, the charge state should be set to 1, or possibly 1 and 2, since the vast majority of metabolites are detected as singly charged ions and only a minority as doubly charged ions. This is in sharp contrast with proteomics applications in which singly charged ions are usually rejected (Kalli et al., 2013).

### 2.8 Rule 8: Perform DDA on all measured samples

A practice sometimes observed in metabolomics studies is to perform full scan measurements with all samples and restrict DDA to only a small subset of samples, for instance on quality control (QC) samples. In our opinion, such approach should be avoided since it may significantly decrease MS/MS coverage by “diluting” each study sample within the pool of QC samples. This is particularly relevant for big sample batches and/or for highly heterogeneous samples. Instead, we recommend to perform DDA on all measured samples to ensure adequate MS/MS coverage.

## 3 CONCLUSION

The last decade of research in tandem mass spectrometry has offered substantial advances for the characterization of the metabolome of biological systems and opened exciting perspectives for a wide range of research areas spread across cellular metabolism, disease detection, drug discovery or biodiversity‐ecosystem function framework (Kaddurah‐Daouk & Krishnan, 2009; Olivon et al., 2017; Withers et al., 2020). Nonetheless, these ever‐growing performances require in‐depth knowledge of the parameters underlying data acquisition processes. DDA methods that potentially offer the highest quality of MS/MS data fully illustrate this strong sensitivity to parameter settings. As highlighted in this tutorial, the inherent nature of DDA imposes important compromises between metabolite screening capacity and the quality of the fragmentation spectra. When developing a DDA method, the trade‐off between quality and quantity must initially be considered since it will strongly impact the data output generated by the processing pipeline. Indeed, while the ability to obtain quantitative and reproducible data for robust comparisons between samples is related to the MS1 screening capacity, the assignment of metabolites using, for instance, molecular networking and in‐silico annotation, is mainly dependent on MS/MS spectral quality. This tutorial provides a practical framework for optimizing the quality of mass spectral fragmentation data while also maintaining efficient screening capacity, thus minimizing the risk of undersampling. Ultimately, this will ensure optimal performance for the use of the last generation processing workflows, including the trinity of feature detection and alignment, molecular networking, and in‐silico annotation (Dührkop et al., 2019; Pluskal et al., 2010; Wang et al., 2016). By improving the accuracy in annotation as well as the clustering capacity of unknown compounds in large sets of metabolites, optimized DDA approaches may provide the finest resolution of the entire metabolomes, and ultimately enhance our ability to explore molecular processes in biological systems.

## CONFLICT OF INTERESTS

Julien Bourquin is an employee of Waters corporation and contributed to this study by helping with the development of DDA methods. Waters had no right of inspection, neither on the conception of this manuscript nor on the decision to publish it.

## Supporting information

Supporting information.
