# Supplementary material for: Eight key rules for successful data‐dependent acquisition in mass spectrometry‐based metabolomics
Source: Mass Spectrom Rev. 2021 Jun 18;42(1):131–43. doi: 10.1002/mas.21715 (PMC10078780; doi:10.1002/mas.21715)
Supplement: Supplementary file 1 — Supporting information. [file MAS-42-131-s001.docx]

**SuppORTING information**

## Eight key rules for successful data-dependent acquisition in mass spectrometry-based metabolomics

Emmanuel Defossez^1^, Julien Bourquin^2^, Stephan von Reuss^3,4^, Sergio Rasmann^1^, Gaétan Glauser^4*^

^1^Laboratory of Functional Ecology, Institute of Biology, University of Neuchâtel, Rue Emile-Argand 11, CH-2000 Neuchâtel, Switzerland

^2^Waters Corporation, Stamford Avenue, Altrincham Road, Wilmslow, SK9 4AX, United Kingdom

^3^Laboratory of Bioanalytical Chemistry, Institute of Chemistry, University of Neuchâtel, Avenue de Bellevaux 51, CH-2000 Neuchâtel, Switzerland

^4^Neuchâtel Platform of Analytical Chemistry, University of Neuchâtel, Avenue de Bellevaux 51, CH-2000 Neuchâtel, Switzerland

^*^ Corresponding author

Gaétan Glauser, Ph.D.

Neuchâtel Platform of Analytical Chemistry

University of Neuchâtel

Avenue de Bellevaux 51

2000 Neuchâtel

Switzerland

Tel: +41327182534

Fax: +41327183001

E-mail: [gaetan.glauser@unine.ch](mailto:gaetan.glauser@unine.ch)

**Method S1**: Example of correct description of a data-dependent acquisition method

Data-dependent acquisition experiments were performed on an Acquity UPLC I-Class (Waters) coupled to a Synapt XS QTOF mass spectrometer (Waters). An Acquity UPLC HSS T3 column (100x2.1mm, 1.8 µm) was used at a flow rate of 0.4 mL/min. The column temperature was maintained at 25 °C. The separation was performed in gradient mode using water and 0.05% formic acid as mobile phase A, and acetonitrile and 0.05% formic acid as phase B. The gradient started at 0% B and increased to 100% B in 12.5 min, followed by a hold at 100% B for 2 min and re-equilibration at 0% B for 4 min. The injection volume was 1 µL using a FTN autosampler and a 10 µL loop.

The Synapt XS was operated in positive electrospray using the high resolution mode (FWHM resolution of 45’000 at *m/z* 556.2766). Data were acquired in continuum mode using the software Masslynx 4.2 (Waters). The capillary voltage was set to 1.0 kV, the cone voltage to 25 V, the source temperature to 140 °C, the desolvation temperature to 500 °C, the desolvation gas flow to 1000 L/h, the cone gas flow to 150 L/h, the nebulizer gas flow to 6.5 bars, and the collision gas (Ar) flow to 2.0 L/min. Full scans were acquired over a mass range of 50-1200 Da with a 100 ms scan time. Every full scan was followed by a maximum of 8 MS/MS of 50 ms each performed on the most intense ions detected in the full scan trace. The threshold for switching to MS/MS was set to 7500 counts per second and the MS/MS selection window was 4 Da. Peak selection was deisotoped and performed only on ions having 1 or 2 charges. After being selected for MS/MS acquisition, the precursor ions were placed on a dynamic exclusion list for 1.5 s. The mass tolerance for exclusion was set to 30 mDa. A static exclusion list of the 100 most intense background ions was generated from a blank sample run just before the batch of samples. Trap and transfer collision energies during the full scan MS were 4 V and 1 V, respectively. For MS/MS, a ramped collision energy of 5-40 V (at *m/z* 50) and 20-70 V (at *m/z* 1200) was applied on the transfer region of the collision cell.

The mass spectrometer was externally and internally calibrated using a 50 µM sodium formate solution and a 0.1 µg/mL leucine-enkephalin (*m/z* 556.2766) solution, respectively.
